# Autonomous Self-Healing Methods as a Potential Technique for the Improvement of Concrete’s Durability

**DOI:** 10.3390/ma16237391

**Published:** 2023-11-28

**Authors:** Anita Gojević, Ivanka Netinger Grubeša, Berislav Marković, Sandra Juradin, Anđelko Crnoja

**Affiliations:** 1 City of Osijek, Franje Kuhača 9, 31000 Osijek, Croatia; anitagojevic@gmail.com; 2 Department of Construction, University North, 104. Brigade 3, 42000 Varaždin, Croatia; acrnoja@unin.hr; 3 Faculty of Dental Medicine and Health, University Josip Juraj Strossmayer of Osijek, Crkvena 21, 31000 Osijek, Croatia; bmarkovic@fdmz.hr; 4 Faculty of Civil Engineering, Architecture and Geodesy, University of Split, Matice Hrvatske 15, 21000 Split, Croatia; sandra.juradin@gradst.hr

**Keywords:** concrete durability, cracks, freezing and thawing, concrete self-healing, autonomous self-healing, bacteria, crystalline hydrophilic additives, capsules

## Abstract

The causes of cracks in concrete are varied, and regardless of their origin, these cracks invariably have a detrimental impact on the durability of concrete structures and escalate their maintenance costs. This paper presents a comprehensive review of current knowledge regarding the methods of self-healing in concrete, ranging from autogenic and improved autogenic self-healing to the autonomous self-healing of concrete. Particular emphasis is placed on the methods of autonomous concrete self-healing: the bacterial healing method, the crystalline hydrophilic additives healing method, and the capsule-based self-healing method. The hypothesis is that applying these self-healing methods could potentially prevent damages or cracks in concrete caused by freeze–thaw cycles, thereby extending the lifespan of concrete structures. The mechanism of action and current achievements in the field are provided for each method.

## 1. Introduction

Cracks in concrete (and in reinforced concrete) are a fairly common occurrence. In specific scenarios, these cracks in concrete do not cause harm and are entirely acceptable. In other instances, cracks are severe defects because they negatively impact the concrete’s strength, function, or appearance. Gardner et al. [1], in their research, state that the occurrence of cracks is one of the primary causes of damage/degradation in structures reported by contractors, designers, and investors. Each year, significant money is allocated globally to repair existing concrete structures. Developed countries, like the USA, Germany, South Korea, etc., face considerable degradation in their concrete structures, leading those countries to spend more on maintenance and repair than on constructing new buildings [1]. According to Danish et al. [2], building maintenance costs are over twice the cost of concrete production, while Du et al. [3] determined that about 50% of construction costs constitute maintenance costs.

Golewski [4] categorizes the causes of crack occurrence into those that appear before and after the concrete has set. Cracks occurring before the concrete has set result from the freezing of young concrete, shrinkage-induced cracking, and those resulting from structural movement during formwork removal. Cracks occurring post hardening are due to physicochemical reactions (alkali-aggregate reaction, steel corrosion), overloading, temperature changes, material fatigue, drying shrinkage, creep, and freeze–thaw cycles. Chemicals, such as acid rains and salts, can easily penetrate these cracks, diminishing their durability. According to Koroth [5], freeze-thaw cycles are a primary factor that decreases material durability. Specifically, water in the material freezes and turns to ice when temperatures drop below freezing, causing the ice, which occupies a larger volume than the original water in a liquid state, to exert pressure on the material’s walls [6]. Repeated freeze–thaw cycles eventually damage the material. For cement composites, these damages manifest either as surface scaling or as internal cracking [7], i.e., the formation of cracks within the cement composite.

A frequent method of crack remediation (whether due to freeze–thaw or other causes) cited by Gardner et al. [1] is repairing the concrete’s protective layer or even strengthening the structure subsequently (for example, with fiber-reinforced polymer (FRP), as presented in [8,9]). However, this is a reactive intervention post damage occurrence; it would be more prudent to address this preemptively at the concrete mix level. In this context, adding air-entraining agents to the mix commonly enhances concrete’s resistance to freeze–thaw cycles [10]. These agents introduce air bubbles into the fresh mix, interrupting the capillaries through which water would ascend into the concrete. Without water in the concrete, freeze–thaw problems are negated. Nevertheless, caution is advised with these agents, as the air bubbles can adversely affect the compressive strength of the concrete. The literature also indicates that concrete resistance can be augmented with mineral additives, such as slag [11], fly ash [12], and silica fume [13]. The porosity and pore distribution in the material significantly affect the resistance of cement composites to freeze–thaw cycles. Zhang et al. [14] have categorized pores directly based on their impact on freeze–thaw resistance as harmless (up to 0.02 µm), less harmful (0.02–0.05 µm), harmful (0.05–0.2 µm), and more harmful (greater than 0.2 µm). Mineral additives are finer than cement, making the cement matrix denser, which likely increases the proportion of smaller and less harmful pores while decreasing the proportion of larger and more harmful ones. Additionally, concrete resistance can also be enhanced by partially substituting aggregate with rubber [15,16,17,18], using polymer binders [19,20], modifying [21,22,23] or impregnating concrete with polymers [24,25], employing polycarbonate superplasticizers [26,27], using biomimetic polymer additives [28], and utilizing polymer fibres [29].

In the study conducted by Gardner et al. [1], nearly one third of the respondents were not satisfied with current approaches taken to address the tendency of concrete to crack, and they expressed their interest in introducing new repair methods in the form of concrete self-recovery. In this context, this paper reviews the current knowledge regarding various self-healing methods for concrete, emphasizing autonomous healing methods that could hypothetically enhance concrete’s resistance to freeze–thaw cycles.

## 2. Autogenous and Improved Autogenous Self-Healing

**Figure 1:**
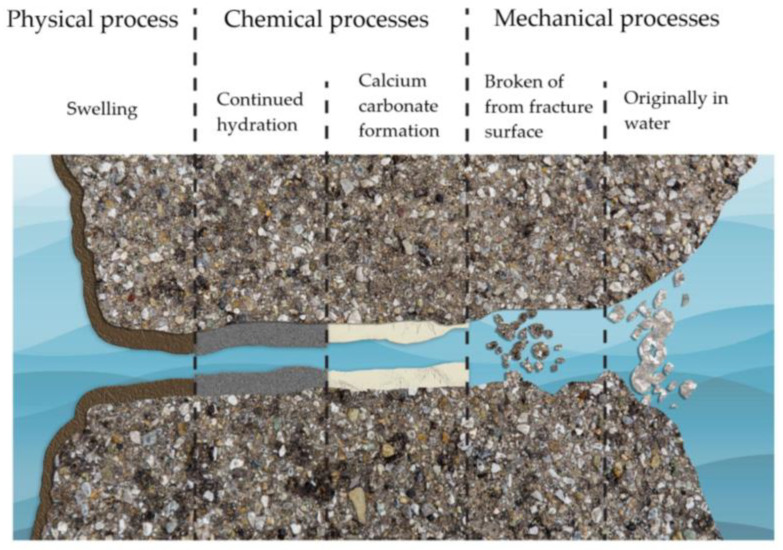
Autogenous healing process.

Autogenous healing is essentially a natural process induced by physical, chemical, and mechanical processes [30,31], as depicted in Figure 1.

### 2.1. Physical Process

Concrete swelling is a slow and partially reversible process caused by water absorption of cement stone. Cracks healed solely by this method may leak again. According to Edvardsen [32], the maximum width of cracks healed by this mechanism is 100 µm when the concrete is continuously in contact with water.

### 2.2. Chemical Processes

Continued hydration is, in fact, the hydration of unhydrated cement particles in concrete, with these unhydrated particles comprising up to 50% of the cement’s mass in conventional concrete [33]. When concrete begins to crack, unhydrated cement particles react with incoming water. This reaction reinitiates the hydration process, creating hydration products that fill the cracks. This healing mechanism is more pronounced in younger concrete than in older concrete. According to Edvardsen [32], this healing mechanism has a minimal effect on the overall healing process. Yuan et al. [34] argue that this healing mechanism is the most effective in the first seven days of healing. The precipitation of calcium carbonate is the primary healing mechanism in autogenous healing. Here, CO_2_ from the air enters the concrete and reacts with H_2_O, forming H_2_CO_3_. Subsequently, ${CO}_{3}^{2-}$ is released and reacts with Ca^2+^ ions in the concrete to form CaCO_3_, which, once its solubility in water is exceeded, begins to deposit along the crack edges, healing them. According to Edvardsen [32], the maximum crack width healed by this mechanism is 200 µm when the concrete is continuously in contact with water.

### 2.3. Mechanical Processes

**Figure 2:**
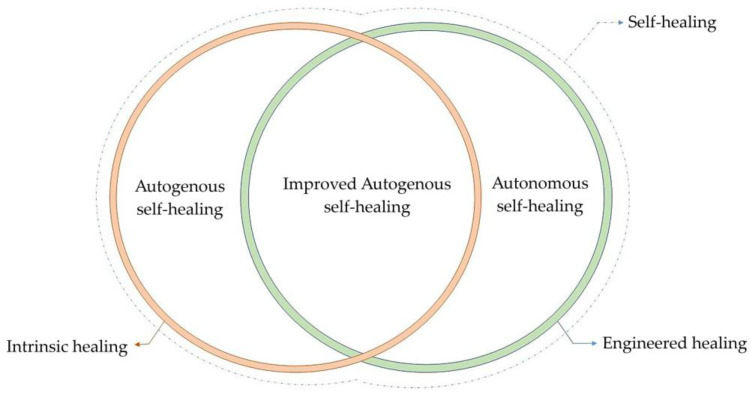
Schematic diagram illustrating self-healing mechanisms [38].

Due to the cracking process of concrete, tiny particles are detached and carried by water through the crack, blocking it and thus participating in the crack healing process. According to Meichsner and Röhling [35], this is effective in the first seven days of healing when the concrete is in contact with water. Key factors affecting autogenous self-healing include the age and composition of the concrete, the presence of water, and the shape and size of the cracks [2]. The age of concrete is crucial for the self-healing mechanism—younger concrete has more unhydrated binding particles available for forming new calcium silicate hydrate (CSH) gel, which is favorable for crack healing. Concerning concrete composition, the clinker content in cement, the silicate content, and the type of aggregate are significant. The clinker content in cement determines the calcium ion supply, ultimately showing the matrix’s ability to form a CaCO_3_ sediment/precipitate. Adding silicates to the concrete mix affects the pozzolanic reaction, the healing process duration, and the consumption of Ca(OH)_2_. The concrete grade is associated with the water-to-cement ratio, the binder’s ability to develop a substantial amount of CSH gel due to hydration, the type of binder, and its amount. Water presence is vital for the autogenous self-healing phenomenon because it enables chemical reactions and it is a transport medium for particles. In this context, water immersion is considered the most appropriate for healing, followed by wet–dry cycles [36]. The latter is feasible because CaCO_3_ can quickly form due to the high availability of carbon dioxide (CO_2_) in the air. The maximum crack width that can be healed by autogenous healing is 150 µm [36] or even up to 600 µm [31]. Although the self-healing process in concrete without human intervention (without any additive) was discovered in 1836 [37], researchers have developed over recent decades various new methods to additionally improve concrete self-healing belonging under categories of improved autogenous or autonomous healing (Figure 2), which are more applicable for the healing of wider cracks.

Improved autogenous healing refers to healing boosted by the addition of fibres (typically polyvinyl alcohol fibres) to the concrete that limit crack width or directly influence the uniform distribution of micro-cracks instead of macro-cracks or by replacing some of the binders with fly ash or slag, which slows down the hydration rate, thus reducing crack formation. Superabsorbent polymers have been reported as effective [38]. In terms of crack healing, metakaolin, limestone, and bentonite have been found effective, along with fly ash [39]. Superabsorbent polymers (SAP) are hydrophilic substances that absorb water when mixed into concrete. As the concrete dries, the SAP releases water back into the concrete, aiding in crack healing [40]. Researchers use SAPs of various origins while varying cement replacement percentages and different particle sizes. Hong et al. [40] used sodium polyacrylate salts of smaller and larger particle sizes in quantities of 0.5% or 1% by cement mass. They found that larger particle SAPs added in higher quantities according to the cement mass were more effective in the crack healing process. Snoeck and De Belie [41] used two types of SAPs: potassium polyacrylates and the copolymer of acrylamide and sodium acrylate. They determined that concrete with added SAP could heal without direct water contact. Zeng et al. [42] improved autogenous healing by adding the surfactant cetyl trimethyl ammonium bromide (CTAB) in quantities ranging from 0.25% to 2.0% to an electrolyte solution (either ZnSO_4_ or MgSO_4_ solution) to improve the crack healing effect through the electrodeposition method. They concluded that both electrolyte solutions were equally effective, and the optimal CTAB amount was 1%, ensuring the most effective healing. Autonomous healing will be the subject of the next section.

## 3. Autonomous or Engineered Self-Healing

Autonomous healing implies healing induced by “artificial means”. Autonomous self-healing techniques include the bacterial method, the application of crystalline hydrophilic additives, and the microencapsulation method [2]. Each of the mentioned methods is discussed in detail below.

### 3.1. Self-Healing of Concrete Using Bacteria

**Figure 3:**
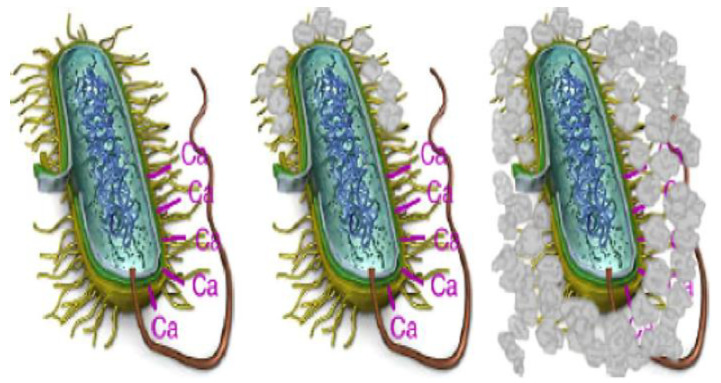
Formation of calcium carbonate on the bacterial cell wall [43].

Certain bacteria added to concrete create urease, which catalyzes urea into carbonate and ammonium, which results in an increase in the pH value of the concrete and an increase in the concentration of carbonate in the bacterial environment. These components further hydrolyze into ammonium (NH^4+^) and carbonate ions CO_3_^2−^, which leads to the formation of calcium carbonate. Figure 3 shows the deposition of calcium carbonate on the bacterial cell wall. The bacterial cell wall is negatively charged, and the bacteria draw cations from the environment, including Ca^2+^, to deposit on the surface of their cell. Ca^2+^ ions react with CO_3_^2−^ in preparation for the deposition of calcium carbonate on the cell surface, which serves as the site of nucleation.

**Figure 4:**
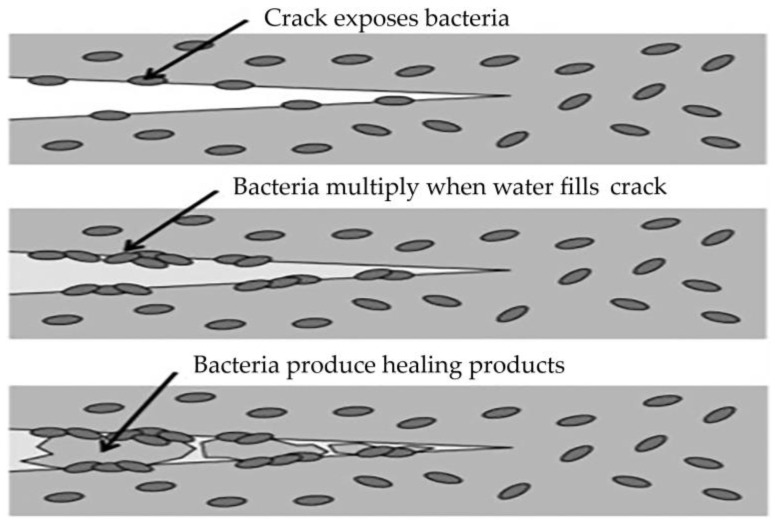
Self-healing process in bacterial concrete [44].

Figure 4 shows a crack created in concrete with embedded bacteria and the process of healing that crack. In fact, bacteria in concrete multiply when a crack occurs and in the presence of water, and calcium carbonate is deposited on their walls, which contributes to the healing of the crack.

The most commonly used bacteria for the purpose of self-healing concrete include *Bacillus subtilis*, *Bacillus cohnii*, *Bacillus pseudomycoides*, *Escherichia coli*, *Bacillus sphaericus*, *Sporosarcina pasteurii*, *Pseudomonas aeruginosa*, and *Diaphorobacter nitroreducens*, or their mixtures. When dosing into concrete mixtures, the authors dose them either directly or through a carrier. In their studies, the authors observe the effect of bacteria on the mechanical and durability properties of concrete and on the self-healing process of concrete. Table 1 shows the types of bacteria used in each study and the main conclusions, while the text below Table 1 gives more details about each study.

Çağatay Erşan et al. [45] investigated the survival ability of unprotected and protected *Pseudomonas aeruginosa* and *Diaphorobacter nitroreducens* bacteria and an unprotected mixture of microbiological cultures containing ACDC. “Protected” refers to bacteria on a carrier (in the form of diatomaceous earth, expanded clay, or granular activated carbon). “Unprotected” refers to bacteria directly introduced into the concrete mix. *Pseudomonas aeruginosa* and *Diaphorobacter nitroreducens* showed a better ability to survive in concrete when protected.

Wang et al. [46], Zhang et al. [47], Zhang et al. [48], and Çağatay Erşan et al. [49], in their research, look at just the influence of bacteria on the self-healing of concrete. In their work, Wang et al. [46] investigated the self-healing of concrete using hydrogels with encapsulated bacterial spores (bio-hydrogels). The bacterial strain used in that study was *Bacillus sphaericus*. Wang et al. [46] made four groups of samples. Group R includes samples without any additives, group N includes samples to which all nutrients were added, including food for bacteria (yeast extract) and precipitation agents (urea and Ca(NO_3_)_2_·4H_2_O), group H includes samples with hydrogel, and group HS comprises samples with hydrogel on which bacteria are encapsulated. A crack was initiated on the concrete cylinders, and they were exposed to an environment of 60% and 95% humidity as well as to dry–wet cycles (1 h in water and 11 h in air at 60% humidity) for 4 weeks. No crack healing was recorded in 60% and 95% humidity, but there was crack healing during dry–wet cycles and in such a way that the best healing was recorded by the group of samples HS, followed by groups H and N, and the lowest healing was recorded by the mixture R. Zhang et al. [47] investigated the potential of the bacterium *Sporosarcina pasteurii* in alkali-activated concrete. They studied the self-healing of alkali-activated concrete with expanded glass granules without bacteria and concrete with bacteria (and calcium lactate and urea as nutrients) introduced into the concrete on a carrier of expanded glass granules. Cracks were initiated on the concrete samples, and their healing was monitored for 90 days. On concrete samples with bacteria (and calcium lactate and urea) introduced on a carrier made of expanded glass granules, healing was monitored in water and during wet–dry cycles. Concrete with bacteria healed better than concrete without bacteria, and healing was more efficient in concrete cured with wet–dry cycles than in concrete cured in water. In another paper, Zhang et al. [48] also studied the self-healing of reference concrete, concrete with *Bacillus cohnii* introduced directly into the concrete, and concrete with *Bacillus cohnii* introduced into the concrete on a carrier made of expanded perlite and expanded clay. The most effective healing was recorded with the mixture with *Bacillus cohnii* on expanded perlite, followed by the mixture with *Bacillus cohnii* on expanded clay. The mixture with *Bacillus cohnii* directly introduced into the concrete healed better than the reference mixture but worse than the mixtures with *Bacillus cohnii* on a carrier of expanded perlite or expanded clay. Microscopic analysis confirmed calcite crystals as healing products. Çağatay Erşan et al. [49] investigated the self-healing ability of concrete with *Pseudomonas aeruginosa* and *Diaphorobacter nitroreducens* on a carrier of expanded clay and granular activated carbon and concluded that both types of bacteria are equally effective on both carriers in the self-healing process of cracked concrete. Microscopic examination of the concrete confirmed CaCO_3_ as a product of crack healing.

In addition to testing self-healing, the influence of bacteria on compressive strength was also tested, and Algaifi et al. [50] compared the compressive strength and self-healing ability of concrete with *Bacillus pseudomycoides* bacteria and control concrete. In order to initiate the self-healing process, a cylindrical sample with a crack was immersed in water at a temperature of 30 °C, which simulates the surrounding tropical temperature with a pH value of water of 7.8. The width of the crack was observed. The results of testing the compressive strength of concrete with bacteria were on average 16% higher than the compressive strength of the control concrete. This is attributed to the microbial deposition of calcium carbonate within the concrete core, as the microbial product filled the microcracks and pores of the concrete. In concrete with bacteria, the crack was completely healed through the deposition of microbes later identified as vaterite and calcite. Safiuddin et al. [51] observed the influence of *Bacillus subtilis* and *Escherichia coli* bacteria separately and in combination on concrete self-healing and compressive strength. Each bacteria was separately dosed into concrete in the amount of 2%, 3%, 4%, and 6% according to the weight of the cement, while in combination (50% each) they were dosed into concrete in the amount of 2% and 3% according to the weight of the cement. For testing compressive strength and monitoring self-healing, cubes were made, and for testing the tensile strength by splitting, cylindrical samples were prepared. It was concluded that cracks were completely healed within 48 h for 2% *Bacillus subtilis*, 32 h for 3% *Bacillus subtilis*, and 72 h for 4% *Bacillus subtilis*. The most favorable proportion of *Bacillus subtilis* bacteria for self-healing concrete would be 3%. *Escherichia coli* bacteria had no effect on self-healing. Separately, each of these bacteria had a positive effect on the development of compressive strength, with the bacterium *Escherichia coli* having a greater impact. Bacteria combined had a negative effect on the compressive strength of concrete. Separately, each of these bacteria had a positive effect on the development of splitting tensile strength, with *Escherichia coli* having a greater influence again. In their paper, Khaliq and Ehsan [52] used *Bacillus subtilis*, which, in the form of a solution, was added directly to the concrete mixture (mixture two), via lightweight aggregate (LWA) soaked in the bacteria solution (mixture three) or via graphite nanoparticles (GNPs) soaked in the bacteria solution (mixture four), while the control mixture was mixture one. The compressive strength with the addition of *Bacillus subtilis* (mixture two) proved to be better at all studied concrete ages than the compressive strength of the control concrete (mixture one). The technique of introducing bacteria using the LWA method (mixture three) proved to be particularly effective. All mixes with bacteria showed better healing over time than the control mix (mixture one). For cracks initiated on the 3rd and 7th day of concrete age, the most effective method was the introduction of bacteria through GNP (mixture four), while in the case of cracks initiated on the 14th and 28th day of concrete age, the most effective method was the introduction of bacteria through LWA (mixture three).

In addition to self-healing and compressive strength, among Pei et al. [53], Jiang et al. [54], Kanwal et al. [55], and Achal et al. [56], some of them also investigate porosity, water permeability, water absorption, and chloride penetration. Pei et al. [53] prepared a control mortar mixture and a mortar mixture with living and dead bacteria and cell walls of living *Bacillus subtilis* bacteria and compared their compressive strengths at the age of 7 and 28 days and their porosity. The results of their test showed that dead and live *Bacillus subtilis* cells had a negative effect on the compressive strength of the cement mortar samples, while *Bacillus subtilis* walls increased the compressive strength by 15%. The increase in the compressive strength of concrete with *Bacillus subtilis* cell walls is a direct consequence of the reduction in porosity of such concrete, and the reduction in porosity directly affects the increase in durability of such concrete. In their research, Jiang et al. [54] saturated expanded perlite (EP) with bacterial solution and wrapped EPs with different coatings (coating made of geopolymers, Portland cement, acid sulfoaluminate cement, potassium magnesium phosphate cement, and hemihydrate gypsum). The resulting coated (and non-coated) EP granules were directly added to the concrete mix. Seven different concrete mixes were prepared: a control mix with non-coated EPs, a mix with non-coated and bacterial-solution-non-saturated EPs, and five mixes with EPs saturated with bacterial solution and coated with different coatings and cracks generated. During the time of 0, 7, 14, and 28 days, the width of the cracks was monitored with a microscope and a ruler, and the water permeability was tested. The results show that bacterial self-healing techniques using EP granules encased in a low-alkaline material, such as geopolymer and Portland cement, significantly improve the ability to heal cracks in concrete, and with increasing healing time, the water permeability coefficient of all samples gradually decreases. Kanwal et al. [55] studied the influence of the bacterium *Bacillus subtilis* (BS) in concrete. They prepared a reference concrete mix, a mix with coal, a mix with BS and calcium lactate as a nutrient, and a mix with coal mixed with BS and calcium lactate as a nutrient. The results show that the addition of BS to the mixture improved the compressive strength of the concrete in all observed periods, and this improvement was more pronounced when BS was immobilized with coal in the mixture. The mixture with BS showed reduced water absorption over time compared to the reference mixture, and the mixture with BS-immobilized coal showed a drastically reduced water absorption. The self-healing of concrete in water with a previously initiated crack was most pronounced in the mixture in which BS was immobilized with coal, and it was slightly less pronounced in the mixture with BS, while the least pronounced was in the reference mixture. SEM, EDS, and XRD analyses confirmed that the healing product is actually CaCO_3_. Achal et al. [56] compared the mechanical and durability properties of a reference mortar and a mortar containing *Bacillus subtilis* (BS). The addition of BS to the mortar increased the compressive strength of the mortar by 40%, reduced the porosity of the mortar by 50%, and significantly reduced chloride penetration and improved the healing of previously induced cracks. Namely, CaCO_3_, which forms in cracks as a self-healing product, is also generally formed in concrete with bacteria and densifies the concrete structure, thus positively affecting the durability properties, and mostly (depending on the type and concentration of the bacteria) also the mechanical properties of the concrete. The increase in the compressive strength of concrete with bacteria is a direct consequence of the decrease in porosity of such concrete, and the decrease in porosity directly affects the increase in durability of such concrete. The lower the water permeability coefficient, the better the effects of self-healing of cracks.

### 3.2. Self-Healing of Concrete Using Crystalline Hydrophilic Admixtures

Crystalline admixtures (CA) are predominantly commercially available products from various manufacturers (e.g., Xypex, Kryton, Penetron, Harbin). They serve a dual purpose: reducing concrete permeability and healing cracks. Their recommended incorporation into concrete ranges from 0.3% to 2% according to the weight of the cement [57]. The physicochemical properties of CA allow them to function both as inert materials and as active chemicals. The European standard EN 934-2 [58] classifies CAs as waterproofing admixtures, specifying three properties to be measured in concretes containing CA to assess their effects and efficiency: capillary absorption, compressive strength, and fresh concrete air content. The American Report on Chemical Admixtures for Concrete, ACI 212.3R-16 [59], categorizes CA under the permeability-reducing admixtures (PRA) subcategory. PRAs are further divided, based on their ability to reduce water ingress under hydrostatic pressure or otherwise, into two subcategories: PRAN (permeability-reducing admixtures submitted to non-hydrostatic conditions) and PRAH (permeability-reducing admixtures exposed to hydrostatic conditions). PRANs are also known for protection against capillary moisture, whereas PRAHs are recognized for waterproofing. PRANs are recommended for delaying, without entirely blocking, the entry and passage of water in liquid or gaseous form under less severe pressure conditions caused by capillary action. Their action renders the concrete surface water repellent or barely wet; thus, a significant contribution of PRANs is the long-term preservation of the aesthetic quality of the concrete, thereby preventing the infiltration of rainwater and groundwater. CAs belong to the PRAH category, indicated for preventing water passage under hydrostatic pressure, thereby reducing concrete permeability and autonomously healing micro-cracks under hydrostatic conditions.

The following four mechanisms describe the self-healing process of concrete with crystalline admixtures [60]:

1. Precipitation Reaction Mechanism—active chemicals penetrate the concrete with water and react with the free lime and oxides in the pores, forming crystalline materials that block pores and cracks. Water is a critical factor stimulating the crystal precipitation in the crack due to the reactive and hydrophilic nature of crystalline admixtures. The reaction between the active compound of the crystalline additive and tricalcium silicate in the presence of water forms a denser calcium silicate hydrate. The effect of the calcium additive can lead to pore clogging, creating a hydrophobic layer in the capillaries, or both. Crystalline additives block pores and, in doing so, deposit hydrates in the cracks to resist water ingress under pressure.
2. Complexation Precipitation Reaction Mechanism—active chemicals bind with Ca^2+^ in concrete, forming an unstable complex that disperses in the pore solution. Complex ions are replaced with SiO_3_^2−^ on non-hydrated cement particles to form C–S–H gels and fill the pores. Active chemicals become free again and continue to diffuse in the solution. The primary identified products of hardened paste are ettringite and calcium silicate hydrate. The primary process for external crack healing is the formation of calcium carbonate, resulting from the action of calcium additives. The interaction of carbonate and bicarbonate ions leads to the precipitation of calcium carbonate, which is associated with increased material durability.
3. Combined Mechanism of Precipitation and Complexation Reactions—part of the active chemicals participates in the capillary crystallization reaction, while another part catalyzes the hydration of non-hydrated cement particles. Limestone formations react with tricalcium aluminate and form different calcium carboaluminates, such as hemi-carbo aluminate, mono-carbo aluminate, and tri-carbo aluminate. Silicate formations (ground quartz) react with calcium hydrate. Limestone formations have a much higher moisture absorption capacity. The high affinity between limestone formation and calcium aluminate favors the crystallization of mono-carbo aluminate over mono-sulfate. This process results in reduced porosity and an increase in the volume of hydrated phases.
4. Condensation Crystallization Mechanism of Active Chemicals—these substances form insoluble crystals through condensation polymerization to fill cracks and pores.

**Figure 5:**
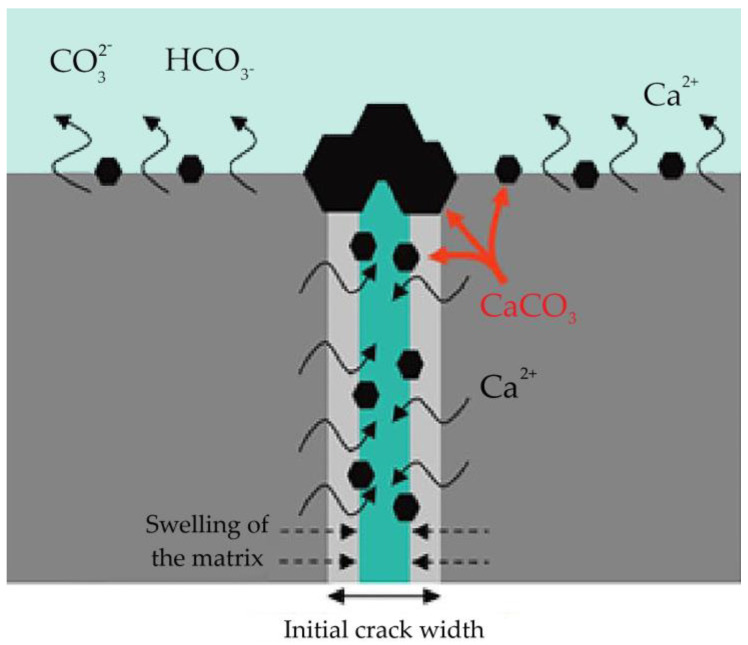
Illustration of calcium carbonate precipitation stimulated by the crystalline admixture [61].

The mechanisms described above are schematically depicted in Figure 5.

In their research, various authors observe the effect of such admixtures on concrete’s mechanical and durability properties and the concrete self-healing process. Table 2 shows the types of CA used in each study and the main conclusions, while the text below Table 2 gives more details about each study.

In the following four studies, Park and Choi [62], Roig-Flores et al. [63], Escoffres et al. [64], and Li et al. [65] examined the effects of self-healing through water flow in samples with an initiated crack. In their research, Park and Choi [62] made six concrete mixtures, with and without a crystalline hydrophilic admixture, and investigated the self-healing ability of such concretes. In concrete mixes, cement was replaced with 35% granulated slag (GGBS) and 5, 7, and 10% expanding agent (CSA). As crystalline hydrophilic additives, they used Na_2_SO_4_ in the amount of 3 or 5% of the cement mass or Al_2_(SO_4_)_3_ in the amount of 3% of the cement mass. Cylindrical samples were made of concrete mixtures, on which a crack was generated, and the samples were placed in a device for measuring the speed of water flow. As the self-healing of the concrete progressed, the rate of water flow decreased. At the same time, the amount of self-healing product was monitored on the crack surfaces. The results show that all mixtures achieved a greater amount of self-healing product per crack and more intense self-healing than the control mixture, and the most effective in self-healing was the combination of CSA with crystalline hydrophilic additives. Roig-Flores et al. [63] investigated the influence of crystalline hydrophilic admixture on the ability of concrete to heal under different conditions. They prepared two concrete mixes, a control mix and a mix in which the filler was replaced with crystalline hydrophilic admixture (CA). After initiation of the crack, the samples were exposed to four different curing regimes for 42 days: continuous immersion in water (WI), contact with water (water height of 2 cm applied to the upper surface of the sample with a crack) (WC), stay in air conditioning chamber with a temperature of 20 °C and 95% humidity (HC), and stay in the laboratory at 40% humidity (AE). The rate of self-healing was calculated from the water flow value in the first measurement and the water flow value after 42 days of healing of the samples. The results show that the highest percentage of healing was achieved if the samples with a crystalline hydrophilic additive were constantly immersed in water, and then if the samples were in contact with water. If the samples stayed in the laboratory space, there was even a further opening of the cracks and an increase in the flow of water. Escoffres et al. [64] investigated the self-healing potential of high-performance cracked concrete with steel fibers (HPFRC) and high-performance concrete with steel fibers and crystalline addition at 2% according to the weight of cement (HPFRC-CA). A three-point bending test was performed on prisms cured in both media (in water and in air), and the mechanical recovery was calculated for each prism as the ratio of tensile stresses on a prism cured for 28 days in a certain medium (water, air) and tensile stresses on cracked prism at the age of 28 days. When treated in air, both groups of prisms achieved the same mechanical recovery, and when treated in water, prisms with crystal additions achieved slightly better recovery than prisms with no crystal additions. SEM analysis showed that calcium carbonate in the form of aragonite formed in the crack of the HPFRC mixture as a self-healing product, while calcium carbonate in the form of aragonite formed in the crack of the HPFRC-CA mixture as a self-healing product. In their paper, Li et al. [65] investigated the effectiveness of different CA coatings in relation to the self-healing ability of concrete. The CA coatings they used were based on sodium carbonate, sodium silicate, sodium aluminate, tetrasodium EDTA, and glycerin. The results showed that concrete with a coating based on sodium silicate had the best self-healing ability. Roig-Flores et al. [66] analyze the self-healing properties of early-age concrete, made with and without the addition of CA (4% according to the mass of cement), by measuring the water permeability of cracked samples and their crack width. Two classes of concrete (C30/37 and C45/55) and three exposure conditions for curing were tested: immersion in water at 15 °C and at 30 °C and wet/dry cycles. The samples were cracked after 2 days of age to a crack width in the range of 0.10–0.40 mm. Three environmental exposure conditions were considered in order to determine the influence of water availability and its temperature on the self-healing ability of the tested samples, thus comparing the reference concrete with the concrete with crystalline admixture. All samples were cured for 42 days under the specified conditions. The best healing ability was shown by concrete samples with CA (for both classes of concrete) cured in water at 30 °C. The samples of the lower class of concrete showed a higher ability to self-heal.

In their paper, Elsalamawy et al. [67] studied the effect of crystal additives in the context of water absorption, and the obtained results were interpreted through SEM and XRD analysis. Twelve concrete mixes were made with three different commercially available crystalline additives (CA) in the amount of 2% according to the mass of cement according to the manufacturer’s recommendation. The results showed that there was a significant decrease in initial water absorption for all samples containing CA compared to the reference mix. The SEM analysis showed a significant decrease in CH content in concrete mixes containing CA, and the XRD analysis showed that crystalline additives of CA materials mainly consist of cement, silicon, and carbonized materials. Lauch et al. [68] investigated the self-healing ability of fiber-reinforced concrete (FRC) containing different admixtures (crystalline admixture CA, expansive agent CSA, and superabsorbent polymer SAP) on samples subjected to different exposure conditions in the laboratory as well as long-term, real-life conditions i.e., outdoor exposure (1 year in Montreal climate). The self-healing ability was evaluated through water permeability testing and macroscopic monitoring of the crack width immediately after the initiation of cracks on the prisms and after the self-healing procedures. The results showed that the control mixture (with no admixtures) performed best in water, then in wet/dry cycles, and worst in air. The self-healing effect of CA is most pronounced in wet/dry cycles, which is further enhanced when CA is added to CSA. The results of the microscopic analysis showed the presence of calcium carbonate in the form of ettringite and homogeneously dispersed calcite in the control mixture and the presence of calcium carbonate in the form of aragonite in the mixture with CA. In their paper, Li et al. [69] investigated the synergistic effect of superabsorbent polymer (SAP) and crystalline admixture (CA) in the amount of 0.2–2% on the cement mass for macrocrack healing in cement-based materials (CBM). The crystalline additives used in this research are citric acid, silica, sodium silicate, sodium carbonate, and a commercial product from the manufacturer Harbin (Harbin CA, Harbin, China). As in [68], the ratio of self-healing was monitored, and it was concluded that of all crystalline additives, citric acid achieves the best synergistic effects with SAP in terms of crack healing.

The following investigations [71,72,73,74,75] observed the effects of self-healing using isothermal calorimetry, where a higher heat of hydration indicated a better ability to further hydrate the material and thus a better ability of that material to self-heal. In their research, Park and Choi [70] used ordinary Portland cement, calcium sulfo aluminate as an expansion agent, and various sulfate-based and carbonate-based crystalline additives. The mixtures with sulfate-based crystalline additives showed a higher heat of hydration than the reference mixture at the age of the samples of 7 days, but as the age of the samples increased (28 and 91 days), the efficiency of the crystalline additives decreased. Mixes with carbonate-based crystalline additives showed a lower heat of hydration than the reference mix at the age of 7 days, but equal or higher heat of hydration than the reference mix at the age of 28 and 90 days. The conclusion of the research is that the effectiveness of the crystalline admixture in terms of the self-healing of concrete depends on the type of admixture (sulphate or carbonate) and the age of the sample at which cracking occurred. Oliveira et al. [71] evaluated healing products during a short time (up to 7 days) and during a longer time (up to 178 days). In order to monitor the healing products during a short time, they made cement pastes from cement and crystal additives (0%, 1%, and 2% cement mass replacement) and mixed it with distilled water and monitored the heat of hydration with an isothermal calorimeter. At this level, it was observed that CA slows down the setting process of the cement paste, and this effect is more pronounced with a higher proportion of CA. SEM analysis established the presence of calcium hydroxide/portlandite (CH) as the primary self-healing product, which confirmed the dual role of CA: CA fills cracks and makes the cement matrix denser.

Reddy and Ravitheja [72] tested the self-healing ability of cracks by testing compressive and tensile strength after the self-healing process. All samples with cracks were treated in one of the following ways to promote concrete self-healing: water immersion (WI), wetting–drying cycles (WD), water contact (WC), and air curing (AE). After 42 days of curing according to a specified regime, they tested the compressive and splitting tensile strengths on such cured samples and compared them to the strengths of concrete where cracks were not initiated. Their research concluded that cracks initiated on the 28th day heal better than those initiated on the 2nd day of the sample age. Water immersion proved to be the most effective for healing all of the curing regimes for samples with cracks, followed by wetting and drying cycles.

Zhang et al. [73] investigated the effects of CA on mechanical and transport properties and the self-healing ability of cement composites. They found that adding CA improved the compressive and tensile strength at the ages of 28 and 56 days. A higher CA content positively impacted compressive strength, and water absorption decreased with increasing CA content in the mixtures. The self-healing ability was observed visually and by tracking water absorption and impermeability in samples with initiated cracks and cracked samples cured for 28 days in water. Regarding self-healing, it was concluded that the best effect was achieved with the highest CA concentration in the mix. Gojević et al. [74] also examined the efficiency of CA on compressive strength, water penetration depth, and the healing ability of concrete with a water–cement ratio of 0.45 and 0.55. The test results showed that adding CA did not affect the compressive strength of both mixes but reduced the depth of water penetration. Mixes with CA addition showed better results in the water penetration depth test for the mixture with a lower water–cement ratio. The crack healing measurements for mixtures with CA were also better than those without CA for both water–cement ratios.

Azarsaa et al. [75] prepared four concrete mixtures using two different types of cement: two mixes without and two with the addition of a crystalline hydrophilic admixture (2% according to the weight of cement). They examined various durability factors. The results showed that mixtures with the crystalline hydrophilic admixture achieved better strengths and lower permeability coefficients than control mixes. However, the study was inconclusive about the specific effect of the crystalline hydrophilic admixture on electrical resistance as a measure of chloride permeability as one of durability properties. Nonetheless, the electric charge and the apparent chloride diffusion coefficient were slightly lower in mixtures with the crystalline hydrophilic admixture, indicating reduced chloride penetration. Based on averaged healing ratios, Azarsaa et al. [75] concluded that the crystalline hydrophilic admixture improved the self-healing of concrete for both cement types.

### 3.3. Self-Healing of Concrete through Capsule Application

**Figure 6:**
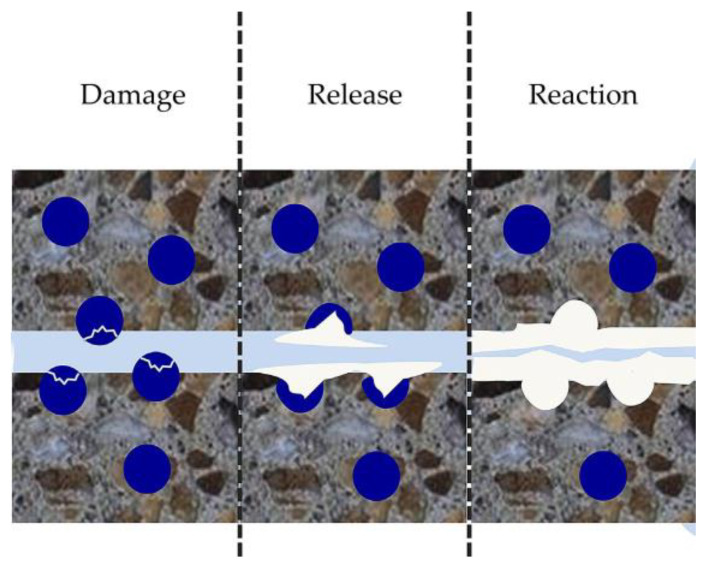
Schematic of mechanically triggered capsule-based self-healing in cementitious matrix [77].

The encapsulation method is considered a versatile and effective strategy for self-healing. In capsule-based self-healing, the capsules provide mechanical protection to healing agents and release them upon being triggered by cracks (either through capsule rupture or diffusion), moisture, air, or changes in the pH of the pore solution in the matrix. When cracking is the trigger mechanism, capsules break, and the healing agent is drawn into the crack via capillary action (Figure 6). Capsule-based healing can be broadly categorized into healing induced by (1) bacterial precipitation and (2) encapsulated chemical healing agents [76]. This section of the paper addresses encapsulation using chemical healing agents, while bacterial precipitation is covered in Section 3.1.

Researchers, in their studies, utilize capsules that are either (1) mixed into the concrete or (2) embedded into previously drilled holes in the concrete or installed in molds/formwork before pouring the concrete mixture. Capsules mixed into the concrete are usually small and are referred to as microcapsules or nanocapsules due to their size, while the size of the latter type of capsule is measured in centimeters. Microcapsules/nanocapsules can be either single-component or multi-component capsules. Table 3 shows the types of capsules used in each study and the main conclusions, while the text below the Table 3 gives more details about each study.

The following four studies show the influence of capsules on crack self-healing. Milla et al. [78] explored the efficacy of microencapsulated calcium nitrate for healing cracks. They produced two types of capsules (with calcium nitrate as the healing agent and urea–formaldehyde as the shell material) with and without emulsifiers (SP with emulsifier, OG without emulsifier) at two different rotation speeds (800 and 1500 rpm). Concrete mixtures were prepared without capsules, with 0.5% and 0.75% capsules. Samples were cracked at an early age (31 days) and cured in water for 7, 21, and 42 days, with crack healing observed using a digital camera. Mixtures with capsules without emulsifiers were more effective at healing, and capsules produced at a higher rpm were more efficient. Wu et al. [79] investigated a dual-component healing system for encapsulating polyurethane and different accelerators based on amine (dimethyl benzylamine, BDMA) and tin (dibutyl tin dilaurate, DBTDL) cast in the mortar. They considered three new encapsulation systems: dual capsules in body contact, parallel-style dual capsules, and concentric-style capsules, all made of glass. Mortar samples were cracked at the age of 28 days, and healing ability was monitored under conditions of 20 °C and 90% relative humidity. Healing efficiency was observed through the radius of the healing agent spread in the concrete, with parallel-style dual capsules showing the highest efficacy. Gilabert et al. [80] examined the crack-filling process via encapsulation. Capsules made from borosilicate glass (3 mm diameter, 175 µm wall thickness, and 50 mm length) were filled with either polyurethane resin or a combination of resin and accelerator (in two separate capsules). These were then embedded into pre-drilled holes in concrete. Cracks were initiated by lightly striking the concrete samples, thus releasing the healing agent. Both capsule types showed that the resin acted more like an adhesive than a healing agent, and the concrete matrix absorbed the accelerator. Hu et al. [81] used a healing agent in the capsules made of polyurethane (PU) diluted with acetone (AC) in varying ratios, with quartz glass forming the capsule shell. Two capsule types were developed: flat-topped capsules and rounded-topped capsules. The former was embedded into pre-drilled holes in the mortar, while the latter was cast in the mortar. It was concluded that AC increased the dispersion area of the healing agent, and the most effective healing ratio was AC: PU at 1:5. Furthermore, rounded-topped capsules were more effective than flat-topped capsules, and two capsules in a mortar sample were more efficient at healing than one, as expected.

In the subsequent three studies, Du et al. [3,82,83] investigated microcapsules using toluene di-isocyanate (TDI) as the core and paraffin, among other additives, as the shell. Du et al. [3,82,83] examined the effects of preparation temperature, ultrasonic mixer rotation speed, ingredient ratios, ambient temperature during self-healing observation of mortar specimens, and various other properties. In their first study, Du et al. [3] prepared microcapsules by initially dissolving 20 g of paraffin at 70 °C, 75 °C, and 80 °C. Subsequently, TDI (with mass ratios of paraffin to TDI being 2:1, 1:1, 1:2, and 1:3) was added dropwise over 30 s, and the paraffin/TDI mixture was stirred for 3 h at speeds of 400, 600, 800, and 1000 rpm. Afterwards, 200 mL of PFTBA cooling agent, an inert liquid that does not react with paraffin or TDI, was introduced under the same mixing conditions to rapidly decrease the paraffin/TDI mixture temperature below paraffin’s melting point. Once the microcapsules, with TDI as the core and paraffin as the shell, were formed in the ultrasonic mixer by stirring, the suspension was poured into a glass and sonicated for 30 min. The microcapsules were filtered out and dried at 40 °C over 24 h. The size distribution and morphology of the microcapsules were characterized using a laser particle size analyzer and scanning electron microscopy (SEM). The optimal preparation parameters for the microcapsules included a paraffin/TDI mass ratio of 1:2 and a stirring speed of 600 RPM at 75 °C, where the microcapsule core’s maximum proportion was 66.5%. FTIR spectra confirmed the successful encapsulation of TDI within the paraffin shell. Five mortar mixtures with varying capsule proportions were prepared. After hardening, the compressive strength was tested, cracks were induced, and the samples were allowed to self-heal for 48 h in air. The best compressive strength and self-healing were achieved with a sample containing 3% microcapsules. In subsequent paper, Du et al. [82] mixed mortar without and with 3% microcapsules, the shells of which were prepared in three distinct ways: paraffin melted at 75 °C and mixed at 600 RPM, paraffin and wax melted at 120 °C and mixed at 800 RPM, and paraffin, wax, and nano SiO_2_ melted at 120 °C and mixed at 800 RPM. The highest compressive strength was observed in the mortar with capsules made of paraffin melted at 75 °C and mixed at 600 RPM. Crack initiation and subsequent healing were monitored in these mortar samples over 1, 3, 7, and 10 days at room temperature. The mortar with the paraffin, wax, and nano SiO_2_ melted at 120 °C and mixed at 800 RPM showed the most successful healing. In final paper by Du et al. [83], cracks were induced in mortars, and their healing was monitored at temperatures of 10, 30, 50, and 60 °C with 50% humidity. Healing was observed through the recovery of compressive strength and in the context of the chloride diffusion coefficient over 1, 3, 7, and 10 days. The researchers concluded that higher temperatures favor crack healing, with 50 °C being the optimal temperature. Healing was most pronounced in mortar mixtures containing microcapsules with a shell of paraffin, wax, and nano SiO_2_ melted at 120 °C and mixed at 800 RPM.

Other researchers [84,85,86,87] have also monitored crack healing in terms of the recovery of compressive strength and in the context of the chloride diffusion coefficient. Li et al. [84] prepared capsules using toluene di-isocyanate (TDI) as the core, with graphite, paraffin, and polyethene wax as the shell. In mortar samples containing 0, 1, 3, 5, 7, and 9% of such capsules according to cement weight and aged 28 days, they determined the compressive strength, the chloride penetration coefficient, and the monitored self-healing on cracked prisms over two curing regimes: room temperature as the first regime and 10 min of microwave treatment, followed by five days of staying at room temperature as the second regime. The results indicate that 5% is the optimal capsule content in terms of the mechanical properties of mortars, and the chloride penetration coefficient also decreases up to a capsule content of 5%. Samples where cracks were induced at 60% of their strength had a higher relative compressive strength than samples cracked at 80%. Regarding the room temperature curing, it proved superior to 10 min of microwave treatment followed by five days at room temperature (the second regime) for the control concrete. However, all concrete with microcapsules achieved better self-healing with 10 min of microwave treatment followed by five days at room temperature. Wang et al. [85] used lightweight aggregate (LWA) as a Na_2_CO_3_ carrier in concrete mixes. They prepared three concrete mixtures, and on the samples they tested the compressive and tensile strength of the samples over time. Chloride penetration and crack healing were initiated at 7 and 28 days of age, and then the samples were immersed in water and a solution saturated with Ca(OH)_2_ for 28 days. The results show that ELWA, impregnated LWA protected by a coating of epoxy resin, curing reagent, and n-butyl glycidyl ether (mixture M3) provides the best compressive and tensile strengths of concrete over time, followed by LWA impregnated with Na_2_CO_3_ (mixture M2). Chloride penetration is the smallest in the mixture where LWA is saturated with Na_2_CO_3_ (mixture M2). The self-healing of cracks is best with the ELWA mixture (Mix M3), followed by the mixture with LWA soaked with Na_2_CO_3_ (Mix M2). Wang et al. [85] came to the conclusion that crack healing is more pronounced in a solution saturated with Ca(OH)_2_ than in water. In their research, Wang et al. [86] produced microcapsules through the polymerization process, i.e., the process of forming microcapsules synthesized using urea–formaldehyde resin as a shell and epoxy resin as a healing agent. They created a control mix of concrete and a mix of concrete with 10% microcapsules per mass of binder in the concrete, and the compressive strength was determined on these samples. The rate of chloride penetration was tested, and their healing was monitored on the initiated cracks. The test results showed that the addition of microcapsules had a negative effect on the 28-day strength of the samples, while the recovery of compressive strength was more pronounced in the mixture with microcapsules as well as the recovery in the context of a 22% lower depth of chloride penetration compared to the same mixture before the treatment procedure. Wang et al. [87] made cylindrical concrete samples with capsules prepared in the previously described manner in the amount of 20% of the volume of the mixture, initiated one or more cracks on them, and exposed the samples wrapped in foil to a temperature of 60 °C. Electrochemical impedance spectroscopy was used to monitor healing after 3, 7, and 14 days, in such a way that a lower resistance reading of the device indicates greater damage. It was concluded that samples with multiple cracks are more prone to self-healing than samples with one crack.

In the papers that follow, Feng et al. [88], Apolinário de Oliveira et al. [89], Papaioannou et al. [90], and Dong et al. [91], in addition to the self-healing, recovery of compressive strength, and chloride penetration tests mentioned so far, also tested waterproofing, electrical resistance, modulus of elasticity, and pore distribution through mercury intrusion porosimetry. Feng et al. [88] investigated mineral incorporation through the encapsulation method using two types of capsules (Type 1 “SC” and Type 2 “WSC”); the basic materials of SC and WSC are “cement + PEG” and “cement + SAP + PEG” (SAP—superabsorbent polymers, PEG—polyethylene glycol) and three types of mortar (control, with SC and WSC capsules). Feng et al. [88] characterized the structure and hydration process of the capsules using SEM and a stereo microscope, the self-healing efficiency of cracked mortars, and the healing product. The results showed that the encapsulated core materials could react with water with the dissolution of PEG and the swelling of crack-swollen SAP; the samples with embedded capsules showed a high sealing ratio for cracks below 400 μm, and even internal cracks spread from 200 μm could be bridged thanks to capsules with SAP. The recovery of water permeability, tensile strength, and compressive strength was achieved, and recovery rates of cracked mortars were higher for mortars containing capsules, especially for SC samples, because SC capsules were more suitable for bridging internal cracks above 200 μm than WSC capsules, while the self-healing products were C-S-H and CaCO_3_. Apolinário de Oliveira et al. [89] compared the mechanical properties, corrosion potential, and self-healing ability of reference concrete (REF), concrete with 3% silica nanocapsules (S3), and concrete with 6% silica nanocapsules (S6). They studied the compressive and tensile strengths of concrete at 28, 90, and 180 days of the sample age on cylinder samples, and the corrosion potential was monitored through electrical resistance on the same type of sample. Cracks were initiated on the 28-day-old samples, and the samples were subjected to a self-healing process lasting 180 days in two different regimes: atmospheric conditions and conditions in a climate chamber (20 °C and 95% humidity). During the self-healing period, tensile strength by splitting, capillary water absorption, and crack healing were monitored on the samples. The results of the test showed that the compressive and tensile strengths of the control concrete were higher during the entire observed period than the compressive strength of the concrete with silica nanocapsules. The electrical resistance of the concrete with silica nanocapsules was higher (and thus less prone to corrosion of the reinforcing steel embedded in such concrete) than the control during the entire observed period. A longer period of self-healing caused a higher level of self-healing, and the least water absorption over time (and thus the highest level of healing) was achieved by concrete samples with silica nanocapsules in the amount of 3% in both curing regimes. Concrete with silica nanocapsules showed complete crack healing after 180 days. Papaioannou et al. [90] produced capsules in which the core was made of Portland cement prepared by pelletizing in a drum. The obtained core was covered with cement in a drum by spraying it with water and a Na_2_SiO_3_ solution to obtain an SS01 capsule shell or only with a Na_2_SiO_3_ solution to obtain an SS02 capsule shell. The capsules thus prepared were added to the mortar by replacing the volume of sand in the amount of 5, 10, and 20%, and prisms were made from the mortar and cured for 28 days in water. Flexural strength, compressive strength, and modulus of elasticity were tested on part of the prisms. A crack was initiated on part of the prisms by applying three-point bending, and they were placed in contact with water to stimulate the self-healing process. To evaluate the self-healing of the mortars, water absorption on the prisms was monitored on the 14th and 28th days, and the water absorption reduction coefficient was calculated from the water absorption. From the obtained results, it is evident that both types of capsules improve the mechanical properties of the mortar and that the SS02 capsules are more successful in this. Furthermore, the water absorption reduction coefficients increase as the proportion of both types of capsules in the mortar increases, and the reduction is greater with the use of SS01 capsules. In their research, Dong et al. [91] made microcapsules from urea–formaldehyde resin as a shell and epoxy resin as a healing agent. They made mortars with 6% of microcapsules on the binder mass with three different mean diameters of microcapsules (132 µm, 180 µm, and 230 µm), from which they made prisms for testing compressive strength, cuboids for mercury porosimetry (MIP), and diameter cylinders for testing the depth of chloride penetration. Cracks were initiated on the prism samples, and they were exposed to curing at 50 °C for 3, 5, 7, 14, and 28 days. Mortar samples with the largest capsules experienced the greatest strength recovery, and this was most pronounced up to the 14th day of curing. MIP confirmed a decrease in the total proportion of pores, pore connectivity, capillary porosity, and mean pore diameter in samples with microcapsules exposed to healing, and by measuring the depth of chloride penetration on samples exposed to healing, it was determined that microcapsules are effective on cracks of all widths.

Hilloulin et al. [92], in their research, focus solely on the properties of capsules, while Van Tittelboom et al. [93], Al-Tabbaa et al. [94], and Araújo et al. [95] investigate not only the properties of the capsules but also their integration into concrete at the structural element level [93,94,95]. Hilloulin et al. [92] examined whether brittle thermoplastics can withstand the concrete mixing process and if they could break upon crack formation at room temperature. They extruded capsules from three different polymers with low glass transition temperatures (Tg): poly(lactic acid) (PLA) (Tg = 59 °C), polystyrene (PS) (Tg = 102 °C), and poly(methyl methacrylate/n-butyl methacrylate) (P(MMA/n-BMA)) (Tg = 59 °C). These chosen polymers (P(MMA/n-BMA), PLA, and PS) exhibit brittle behavior at room temperature during concrete mixing but behave much better when heated before use in concrete. Such capsules have a higher survival rate than glass capsules, but heating polymer capsules complicates the process of self-healing concrete. Thus, exploring additional methods to enhance their ductility is essential. Van Tittelboom et al. [93] monitored the self-healing behavior of concrete at the reinforced concrete beam level produced from three different concrete mixes: control concrete (REF), concrete combined with capsules having a polyurethane core and glass shell (PU), and concrete containing superabsorbent polymers (SAP). The reinforced concrete beams were subjected to four-point bending to initiate cracks, followed by water spraying four times daily for one minute over six weeks to promote crack self-healing. The results showed that the superabsorbent polymer was most effective for healing cracks of all widths. Polyurethane capsules were most effective for the most expansive cracks but the least practical for the narrowest ones. Al-Tabbaa et al. [94] utilized microcapsules with a shell made of gelatin/gum Arabic, and the healing agent was sodium silicate emulsified with mineral oil and an emulsifier. Microcapsules were added to the concrete mix at 2.67% of the cement weight. Walls were cast from this concrete, and a wall was constructed from reference concrete. Hydraulic presses initiated a crack of 0.5 m from the bottom of both walls, and their healing was monitored over time. It was observed that after the self-healing period, the wall containing microcapsules had a significantly lower permeability coefficient, a much higher percentage of crack healing (both in width and depth), and a significantly higher strength recovery compared to the reference concrete wall. Araújo et al. [95] initially assessed the suitability of polymeric cylindrical capsules made from poly(methyl methacrylate) (PMMA) against glass capsules for carrying healing agents in concrete and the parameters affecting the survival rate of these capsules during fresh concrete mixing. They considered five different capsule treatments. Capsules with wall thicknesses of 0.2 mm and 0.4 mm did not survive the concrete mixing process; hence, only capsules with a 0.7 mm wall thickness were chosen for further testing. Mortar samples were then prepared with a single capsule positioned roughly 1.3 cm from the bottom, upon which a crack was initiated. Araújo et al. [95] referred to the healing agent inside the capsule as a water-repellent agent (WRA). The healing efficiency post crack was assessed using a capillary water absorption test. The results clearly showed that the water ingress was lesser for self-healing cracked concrete samples with capsules than those without. Glass and PMMA capsules were equally effective. They produced three real-sized concrete beams to validate the healing effectiveness in more prominent concrete elements; one contained PMMA capsules, the other contained glass capsules, and the third one was a reference one without capsules. The self-healing efficacy of encapsulation materials (glass or PMMA) was assessed, revealing that cracked concrete beams containing capsules (either glass or PMMA) filled with a water-repellent agent exhibited much better resistance to chloride penetration than standard cracked concrete beams. However, PMMA capsules showed lower self-healing efficiency (regarding chloride penetration) than glass capsules due to their less uniform distribution in the concrete. Furthermore, concrete containing glass capsules is sensitive to alkali–silica reactions. Although further optimization of PMMA capsules is necessary to improve their distribution in concrete and achieve higher self-healing efficiency, the results obtained by Araújo et al. [95] indicate that these capsules might be a promising solution for self-healing concretes.

Developing suitable capsules is paramount for achieving encapsulated self-healing. In the context of self-healing concrete, capsules that can easily be mixed into the concrete and release the healing agent upon cracking are ideally required. Optimizing these properties would enable successful large-scale implementation in practical applications [94]. Microcapsules can encapsulate limited amounts of repair agents, and most of the healing agent is exhausted in one healing cycle, making repeated healing over an extended period questionable. Hence, recent research efforts have been directed towards the smart release of healing agents.

## 4. Conclusions

This study systematically presents the state of the art of methods for concrete self-healing while emphasizing autonomous self-healing (healing by bacteria, healing through crystalline hydrophilic additives, and healing via capsules). It evaluates the diverse properties of concrete both before and after the self-healing process. The mentioned methods could serve as an intelligent approach to enhance the concrete’s resistance to freeze–thaw cycles and extend the lifespan of structures. Taking action during concrete mix preparation for incorporation into structures makes it possible to minimize damages/cracks resulting from freeze–thaw cycles, thereby significantly reducing the inspection labor and maintenance costs of such structures [96]. The application of these self-healing concretes is foreseen in structures that are challenging to access for repairs, such as bridges, water reservoirs, structures prone to chemical reactions, pre-fabricated tunnel sections, tunnel connections, nuclear facilities, dams, concrete roadways, pylons, and airstrips.

## Figures and Tables

**Table 1:** Types of bacteria used in concrete and their effects on concrete properties.

Authors	Type of Bacteria	Tested Properties	Results
Çağatay Erşan et al. [45]	Protected and unprotected *Pseudomonas aeruginosa* and *Diaphorobacter nitroreducens*; unprotected mixtures of microbiological cultures containing activated compact denitrification core (ACDC)	Survival in concrete	Unprotected mixture of microbiological cultures containing ACDC was superior; *Pseudomonas aeruginosa* and *Diaphorobacter nitroreducens* survival was better when protected
Wang et al. [46]	*Bacillus sphaericus* with nutrients; hydrogel; hydrogel encapsulated bacteria; no bacteria	Crack healing in different humidity conditions	Best healing during wet–dry cycles for specimens with hydrogel encapsulated bacteria
Zhang et al. [47]	*Sporosarcina pasteurii* on expanded glass granules; expanded glass granules	Crack healing in water and wet–dry conditions	Better healing during wet–dry cycles for specimens with bacteria on expanded glass granules
Zhang et al. [48]	*Bacillus cohnii* directly added; *Bacillus cohnii* added on a carrier made of either expanded perlite or expanded clay; no bacteria	Crack healing in water	Best healing for specimens with bacteria on expanded perlite
Çağatay Erşan et al. [49]	*Pseudomonas aeruginosa* and *Diaphorobacter nitroreducens* added on a carrier of either expanded clay or granular activated carbon	Crack healing in water	Both types of bacteria were equally effective on both carriers
Algaifi et al. [50]	*Bacillus pseudomycoides*; no bacteria	Crack healing in water; compressive strength	Healing was better and compressive strength was higher for concrete with bacteria
Safiuddin et al. [51]	*Bacillus subtilis* and *Escherichia coli* each separately and combined	Crack healing by sprinkling water; compressive strength; splitting tensile strength	*Bacillus subtilis* positively influenced healing; each bacteria separately had a positive effect on compressive and splitting tensile strength but a negative effect when combined
Khaliq and Ehsan [52]	*Bacillus subtilis* added either directly or on a carrier of lightweight aggregate/graphite nanoparticles; no bacteria	Crack healing in water; compressive strength	Graphite nanoparticles were a more efficient carrier; bacteria improved compressive strength regardless of whether it is applied
Pei et al. [53]	Living and dead *Bacillus subtilis* bacteria and cell walls of living *Bacillus subtilis*	Compressive strength	Dead and live *Bacillus subtilis* cells had a negative effect on compressive strength; *Bacillus subtilis* walls increased compressive strength
Jiang et al. [54]	*Bacillus cohnii* added on a carrier made of expanded perlite non-coated or coated with geopolymers, Portland cement, acid sulfoaluminate cement, potassium magnesium phosphate cement, and hemihydrate gypsum; no bacteria	Crack healing in water	Geopolymer and Portland cement coatings significantly improved healing
Kanwal et al. [55]	*Bacillus subtilis* with or without coal; no bacteria	Crack healing in water; compressive strength; water absorption	Best healing for bacteria with coal; bacteria with or without coal improved compressive strength and reduced water absorption
Achal et al. [56]	*Bacillus subtilis*; no bacteria	Crack healing in contact with water; compressive strength; porosity; chloride penetration	Bacteria improved healing, increased compressive strength, reduced porosity, and reduced chloride penetration

**Table 2:** Types of CA used in concrete and their effects on concrete properties.

Authors	Type of Crystalline Hydrophilic Admixture (CA)	Tested Properties	Results
Park and Choi [62]	Na_2_SO_4_ or Al_2_(SO_4_)_3_, with or without expanding agent (CSA); no CA	Crack-healing in contact with water	CA promotes healing, especially when combined with CSA
Roig-Flores et al. [63]	Unnamed CA; no CA	Crack healing in different humidity conditions	CA promotes healing, especially when specimens are in contact with water
Escoffres et al. [64]	Sika WT-250; no CA	Mechanical recovery in terms of bending strength of specimens cured in water and in air	CA slightly improved recovery when specimens were in water
Li et al. [65]	CA coatings based on sodium carbonate, sodium silicate, sodium aluminate, tetrasodium EDTA, and glycerin	Crack healing in contact with water	Coating based on sodium silicate had the best healing ability
Roig-Flores et al. [66]	Unnamed CA; no CA	Crack healing in different humidity conditions	CA promotes healing, especially when specimens are cured in water at 30 °C
Elsalamawy et al. [67]	Three different commercially available CAs; no CA	Initial water absorption	CA significantly reduces initial water absorption
Lauch et al. [68]	Penetron admix alone or combined with expansive agent (CSA)/superabsorbent polymer (SAP); no admixture	Crack healing in different humidity conditions	CA promotes healing, especially in wet/dry cycles, which is further enhanced when CA is combined with CSA
Li et al. [69]	Citric acid, silica, sodium silicate, sodium carbonate, and a commercial product from the manufacturer Harbin, all combined with SAP	Crack healing in contact with water	Citric acid achieved the best synergistic effects with SAP in terms of crack healing
Park and Choi [70]	Various sulfate-based and carbonate-based CAs; no CA	Heat of hydration	Sulfate-based CA promoted healing at an early age, while carbonate-based CA promoted healing at a later age
Oliveira et al. [71]	Unnamed CA; no CA	Heat of hydration	CA slows down the setting process of the cement paste
Reddy and Ravitheja [72]	Unnamed CA	Mechanical recovery in terms of compressive strength and split tensile strength of specimens cured in different humidity conditions	Water immersion of specimens best promotes healing
Zhang et al. [73]	CA made of ion chelator, calcium formate, silica sol, and ethylene–vinyl acetate; no CA	Crack healing in contact with water; compressive strength	CA positively impacted healing and compressive strength
Gojević et al. [74]	Penetron admix; no CA	Crack healing in water; compressive strength; water penetration depth	CA improved healing, had no effect on compressive strength, and reduced water penetration depth
Azarsaa et al. [75]	Unnamed CA; no CA	Crack healing in contact with water; water penetration depth; electrical resistivity; resistance to chloride penetration	CA improved healing, reduced water penetration depth, had no effect on electrical resistivity, and improved resistance to chloride penetration

**Table 3:** Types of capsules used in concrete and their effects on concrete properties.

Authors	Capsule Types	Tested Properties	Results
Milla et al. [78]	Microcapsules made of calcium nitrate as a healing agent and urea–formaldehyde as a shell material, with and without emulsifiers added; no microcapsules	Crack healing in water	Microcapsules improved healing; the ones without emulsifiers were more effective in healing
Wu et al. [79]	Glass capsules with a dual-component healing system for encapsulating polyurethane and different accelerators	Crack healing in air	Polyurethane is a very effective healing agent
Gilabert et al. [80]	Capsules made of borosilicate glass filled with either polyurethane resin or a combination of polyurethane resin and accelerator	Crack healing in air	Polyurethane resin acted more like an adhesive than a healing agent
Hu et al. [81]	Capsules made of quartz glass filled with polyurethane as a healing agent diluted with acetone	Crack healing in air	Acetone increased the dispersion area of the healing agent
Du et al. [3]	Microcapsules made of toluene di-isocyanate as a core and paraffin as a shell; no microcapsules	Crack healing in air; mechanical recovery in terms of compressive strength	Microcapsules promoted healing and mechanical recovery
Du et al. [82]	Microcapsules made of toluene di-isocyanate as a core and paraffin/paraffin with wax/paraffin with wax and nano SiO_2_ as a shell; no microcapsules	Crack healing in air	Microcapsules with a shell made of paraffin with wax and nano SiO_2_ showed the most successful healing
Du et al. [83]	Microcapsules made of toluene di-isocyanate as a core and paraffin/paraffin with wax/paraffin with wax and nano SiO_2_ as a shell; no microcapsules	Mechanical recovery in terms of compressive strength at different temperatures; recovery rate of chloride diffusion coefficient	Higher temperatures favored crack healing
Li et al. [84]	Microcapsules made from toluene di-isocyanate as a core, with graphite, paraffin, and polyethene wax as a shell; no microcapsules	Crack healing in two curing regimes: room temperature and 10 min of microwave treatment followed by five days at room temperature; compressive strength; chloride diffusion coefficient	Ten min of microwave treatment followed by five days at room temperature ensured better healing than room temperature curing; 5% of microcapsules improved compressive strength and reduced chloride diffusion coefficient
Wang et al. [85]	Lightweight aggregate (LWA) as a Na_2_CO_3_ carrier in concrete mixes (coated and non-coated); lightweight aggregate (LWA)	Crack healing in water and in a solution saturated with Ca(OH)_2_; compressive and tensile strength; chloride penetration coefficient	Healing was better in a solution saturated with Ca(OH)_2_; coated LWA ensured the best healing and the highest compressive and tensile strength; non-coated LWA ensured the lowest chloride penetration coefficient
Wang et al. [86]	Microcapsules synthesized using urea–formaldehyde resin as a shell and epoxy resin as a healing agent; no microcapsules	Crack healing at room temperature; recovery in terms of compressive strength and chloride penetration coefficient	Microcapsules improved healing and had a positive effect on recovery in terms of compressive strength and chloride penetration coefficient
Wang et al. [87]	Microcapsules synthesized using urea–formaldehyde resin as a shell and epoxy resin as a healing agent; no microcapsules	Crack healing at room temperature of single-cracked specimens and multiple-cracked specimens	Samples with multiple cracks are more prone to healing than samples with one crack
Feng et al. [88]	Capsules made of cement and polyethylene glycol (PEG) or cement, superabsorbent polymer (SAP), and polyethylene glycol (PEG); no capsules	Crack healing in water; recovery in terms of compressive and flexural strength as well as water permeability	Capsules improved healing and recovery in terms of compressive and flexural strength as well as water permeability; capsules with SAP were more efficient
Apolinário de Oliveira et al. [89]	Nanocapsules made of silica; no nanocapsules	Crack healing at room temperature and in high-humidity chamber; compressive and tensile strength; electrical resistance	Healing was better in high humidity chamber; microcapsules improved healing efficiency and decreased compressive and tensile strength but increased electrical resistance
Papaioannou et al. [90]	Capsules made of Portland cement prepared by pelletizing in a drum as a core and Na_2_SiO_3_ solution as a shell; no capsules	Healing in contact with water; flexural and compressive strength; modulus of elasticity	Capsules improved healing, compressive strength, and modulus of elasticity but reduced flexural strength
Dong et al. [91]	Microcapsules from urea–formaldehyde resin as a shell and epoxy resin as a healing agent; no microcapsules	Recovery in terms of compressive strength, water permeability, and chloride penetration depth	Microcapsules positively influenced recovery of compressive strength, water permeability, and chloride penetration depth
Hilloulin et al. [92]	Extruded capsules from different polymers	Survival in concrete	Capsules exhibited brittle behavior during concrete mixing
Van Tittelboom [93]	Capsules with polyurethane core and glass shell (PU); no capsules	Healing through water spraying	Capsules were most effective for the most expansive cracks
Al-Tabbaa et al. [94]	Microcapsules with a shell made of gelatin/gum Arabic and sodium silicate as a core; no microcapsules	Healing in contact with water; strength recovery	Microcapsules improved healing and strength recovery
Araújo et al. [95]	Capsules made of water-repellent agent as a core and polymeric cylindrical capsules (PMMA) or glass capsules as a shell; no capsules	Healing in contact with water	PMMA and glass capsules improved healing process

## Data Availability

The data presented in this study are available upon request from the corresponding author.
